# A Manual of Ausculation and Percussion

**Published:** 1886-10

**Authors:** 


A MANUAL OF AUSCULTATION AND PERCUSSION; EMBRACING THE PHYSICAL DIAGNOSIS OF DISEASES OF THE LUNGS AND HEART, AND OF THORACIC ANEURISM. *By* AUSTIN FLINT, M. D., LL. D., *Professor of the Principles and Practice of Medicine and of Clinical Medicine in the Bellevue Hospital Medical College, etc., etc. Fourth Edition, thoroughly Revised and Enlarged. Illustrated with fourteen woodcuts. Duodecimo, pp. xii, 280. Philadelphia:* LEA BROTHERS & CO. *Chicago:* JANSEN, MCCLURG & CO., *1885.*

This manual no longer requires extended notice. The celebrity of its author and the merit of previous editions assures for it continued recognition as an authority on the subjects of which it treats. The different physical signs, together with the normal and pathological conditions on which they depend, are first described, and then the various diseases of the chest are discussed in relation to differential diagnosis by physical examination; an arrangement which furnishes to the practitioner a means of ready reference, and to the student a useful reiteration of the contents.

Discussion of doubtful points in the mechanism of signs is expressly waived. This is very well for the sake of brevity, but unsatisfactory in other particulars. One naturally inquires

*why* a vesiculo-*tympanitic* resonance is obtained by percussing over the condensed lung above a pleuritic effusion.

The book is written in a pleasant colloquial style, calculated to enhance the interest of the reader, while at the same time the phraseology is clear and concise. W. E. C.

